# The treatment zone size and its decentration influence axial elongation in children with orthokeratology treatment

**DOI:** 10.1186/s12886-021-02123-x

**Published:** 2021-10-12

**Authors:** Weiping Lin, Na Li, Tianpu Gu, Chunyu Tang, Guihua Liu, Bei Du, Ruihua Wei

**Affiliations:** 1 Eye Institute and School of Optometry, Tianjin, China; 2 Tianjin Medical University Eye Hospital, Tianjin, China; 3 Tianjin Key Laboratory of Retinal Functions and Disease, Eye Institute and School of Optometry, Tianjin Medical University Eye Hospital, Tianjin, 300384 China

**Keywords:** Myopia control, Orthokeratology, Treatment zone size, Treatment zone decentration

## Abstract

**Background:**

To investigate whether the treatment zone size (TZS) and treatment zone decentration (TZD) will affect the axial elongation in myopic children undergoing orthokeratology treatment.

**Methods:**

A self-controlled retrospective study was conducted on 352 children who met the inclusion criteria. Axial length was measured before and at 12 months after the initial lens wear. Corneal topography was measured at baseline and at each follow-up after lens wear. The Corneal topography obtained from the 12-month visit was used to quantify TZS and TZD for each subject. Cycloplegic refraction was required for all children before fitting the orthokeratology lenses.

**Results:**

Axial elongation was significantly associated with age, baseline spherical equivalent (SE), TZS, and TZD with univariate linear regression. In groups with both small and large TZS, axial elongation was significantly decreased with large TZD (both *P* < 0.01). In groups with both small and large TZD, axial elongation was significantly decreased with small TZS (*P* = 0.03 for small TZD, *P* = 0.01 for large TZD). Age, SE, and TZD were significantly associated with axial elongation in multiple regression (all *P* < 0.01).

**Conclusion:**

Relatively smaller TZS and larger TZD may be beneficial in slowing myopia progression in children with orthokeratology treatment.

## Background

Myopia has become a serious public health issue due to its increased prevalence and severity over the past decades [1, 2]. Myopia has increased in prevalence to 20–30% in western countries and 40–70% in Asian populations [3, 4]. In East Asian countries, 80% of 18-year-olds are myopic [5]. Progression of myopia, with axial elongation and eyeball expansion, increased the risk of a series of myopia pathological changes, such as macular degeneration, posterior scleral staphyloma, and choroidal neovascularization [6, 7]. Therefore, prevention and control of myopia in children has become a topic of significant interest.

A variety of methods have been used for controlling the progress of myopia, including orthokeratology, which has an effectiveness supported by several studies [8–11]. An orthokeratology lens is a rigid contact lens with a reverse geometry on its back surface [12]. Through overnight wearing, the central portion of the cornea is flattened to correct refractive errors for good daytime vision. Relative corneal refractive power in the mid-peripheral cornea is increased, inducing myopic defocus on the peripheral retina. Animal studies have demonstrated strong inhibitory effects of peripheral myopic defocus on axial elongation or myopia development [13–15]. It has been proposed that changes in peripheral retina defocus [16–18] and aberrations [11, 19, 20](especially spherical aberrations and vertical coma) may be responsible for the reduced myopia progression reported with orthokeratology.

A series of factors have been proposed to be associated with the individual variation in axial elongation with orthokeratology, such as age, baseline spherical equivalent (SE), corneal shape, and pupil size [21–23].Recently, a study reported a negatived correlation between treatment zone decentration (TZD) and axial elongation [24]. However, it is not clear how decentration contributes to the slowing of myopia progression, but factors such as increased corneal coma [11, 19, 25, 26],and increased corneal asymmetry [22]have been suggested. Another factor, treatment zone size (TZS), was also suggested to be associated with slowing myopia progression [27–31].

The mechanism by which TZS and TZD affect myopia control may be associated with the distribution of the corneal power shift or the conditions of the retinal peripheral defocus. To our knowledge, few study to date has combined these two factors in an orthokeratology study. The purpose of this current study was to explore the effect of TZS combined with TZD on slowing myopia progression in children using orthokeratology. This will enhance our understanding of the effects of orthokeratology in slowing myopia progression over a topography profile.

## Methods

### Subjects

This retrospective study was conducted at the Tianjin Medical University Eye Hospital (Tianjin, China) between May 2018 and July 2019. This study adhered to the tenets of the Declaration of Helsinki and was approved by the Ethics Committee of Tianjin Medical University Eye Hospital. In total, 352 children were deemed suitable for this study and included for analysis according to the inclusion criteria below. The patients were recruited from the children who visited the myopia control clinic. The initial inclusion criteria for orthokeratology lens fitting were: aged between 8 and 14 years; SE of cycloplegic refraction from − 0.75 D to − 6.00 D; corneal astigmatism ≤1.50 D; best-corrected monocular visual acuity no less than 20/20; cycloplegic refraction with compound tropicamide eye drops (5 mg/mL, one drop every 5 min for four times) before fitting orthokeratology lenses. Exclusion criteria were: strabismus or ocular surface disease; any history of surgery or contact lens use; binocular vision dysfunction.

### Orthokeratology lens fitting and follow up plan

Children were fitted with spherical four-zone orthokeratology lenses (Euclid Systems Corporation, Herndon, USA) composed of oprifocon A (Boston EQUALENS II) with an oxygen permeability of 127× 10^− 11^ (cm^2^/s) (mL O_2_/mL · mm Hg). Total lens diameter had a range of 10.2–11.0 mm. Lens fitting procedures strictly followed the guidelines provided by the lens manufacturer. Briefly, the first trial alignment curve for the lens was based on the corneal topography (Medmont, International Pty. Ltd., Victoria, Australia), flat-K, corneal eccentricity, and horizontal visible iris diameter. Fitting quality was evaluated by fluorescence staining 1 h after the lens placement. A good fitting was indicated by an optical zone covering the pupil, no apparent decentration of the lens, blink lens-movement less than 1 mm, and a bullseye pattern with fluorescence staining. Over-refraction was performed to determine target power plus 0.75 diopters as the final order. Children received instructions for contact lens wearing and cleaning at fitting. Lenses were required to be worn for more than 8 h per night, for at least 6 days per week. Follow-up visits were scheduled at one day, one week and one month after the initial lens wear, and at least once every 3 months afterward. All children included were continuously worn the lenses and do topographic map examination within four hours after removing the lenses. A total of 352 children meet the above conditions.

### Determination of treatment zone size and treatment zone decentration

**Fig. 1:**
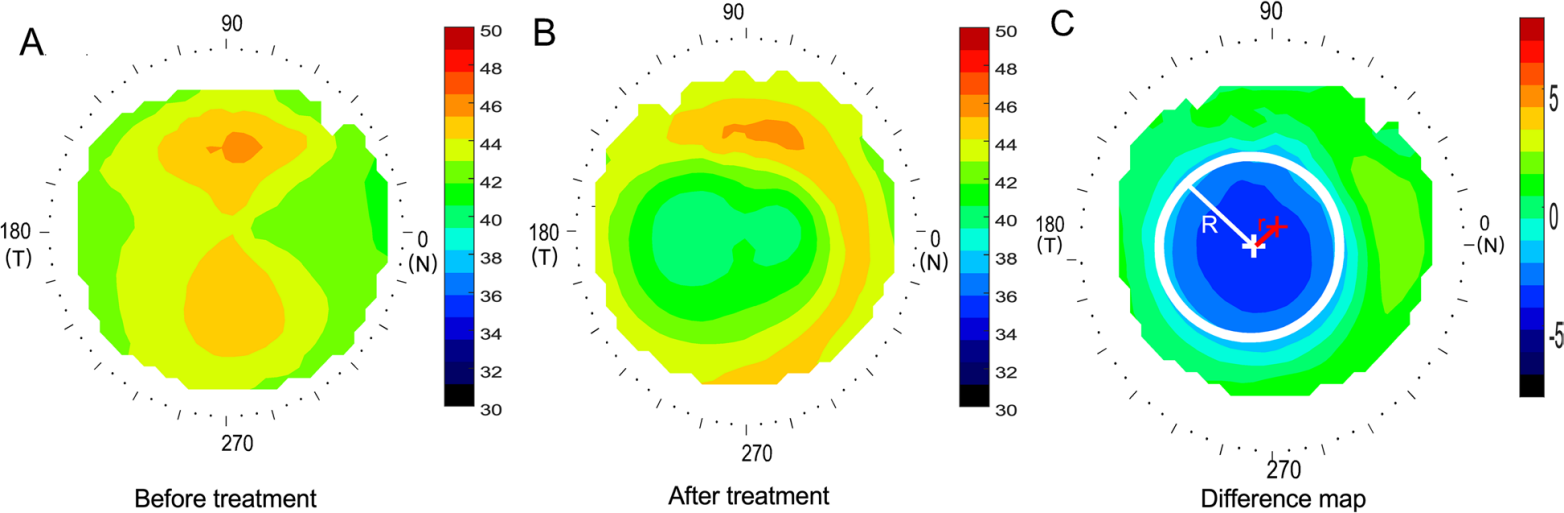
Methods to determine the treatment zone decentration and treatment zone size. **A** Axial map at baseline; **B** tangential curvature map at the twelve-month visit; **C** difference map used to determine treatment zone decentration and treatment zone size. The red cross indicates the corneal apex, the white circle represents the fitted treatment zone, and the white cross indicates the center of the treatment zone. The distance between the white cross and red cross was defined as the decentration of the treatment zone. The radius of the white circle was defined as the treatment zone size. R: radius of treatment zone circle; r: the distance of treatment zone decentration

Corneal topography was first obtained with a Medmont Corneal Topographer at baseline (Fig. 1A). Three corneal topography maps were performed at each follow-up visit and each of the profiles used was the best-focused image (with and accuracy of > 95%) of the frames that were captured automatically. The topography parameters were computed based on the topography obtained 12 months after the initial lens wear (Fig. 1B). To determine the central treatment zone, a difference map was calculated by subtracting the post-treatment tangential curvature map from the baseline map. The area containing locations reduced by more than 0.00 D was defined as the treatment zone, and its boundary was fitted to a circle using a custom Matlab function (MathWorks, Natick, WA) (Fig. 1C). The center of the circle was defined as the center of the treatment zone (white cross, Fig. 1C). In previous study [32], the corneal center after Ortho-k treatment was less than 0.1 mm on average by comparing the apex position relative to the pupil center, then the distance between the center of the circle and the geometric center of the corneal (red cross, Fig. 1C) was defined as the TZD (r, Fig. 1C). The radius of the fitted circle (R) was defined as the TZS (R, Fig. 1C). This method to quantification of TZD relative to the corneal center had been used in previous paper [33].

### Axial length measurement

Axial length was measured before lens fitting (baseline) and at 12 months after lens treatment using noncontact optical biometry (Lenstar 900; Haag-Streit AG, Switzerland). At each visit, a single examiner measured the axial length three consecutive times, and the mean value was taken for data analysis. All measurements were done by the same experienced technician, and the results meet the quality control requirements of the instrument.

### Statistical analysis

For descriptive purposes, the means and standard deviations were calculated for baseline SE, ages, axial elongation and corneal parameters (flat-K, steep-K). The normality of the data was tested with a Schapiro-Wilk test. Univariate linear regression was used to analyze the relationships between axial elongation and age, baseline SE, TZS, TZD. Stepwise multiple linear regression was used to analyze the relationships between axial elongation and age, baseline SE, TZS, TZD and corneal parameters. All statistical analyses were performed using R software (version 3.2.2 http://www.R-project.org/). A *P* < 0.05 value was defined as statistically significant.

## Results

### Measurements

Corneal topography data were retrospectively collected from the clinical records of the 352 children; only the right eye data (352 eyes) was used for statistical analysis. The mean age of the subjects was 10.28 ± 1.88 years (range 8–14 years). At baseline, the mean SE was − 3.25 ± 1.28 D (range − 6 to − 0.75 D), the mean axial length was 24.92 ± 1.36 mm, the mean corneal flat-K was 42.65 ± 3.88 D, the mean corneal steep-K was 43.88 ± 3.96 D. After treatment, the mean axial elongation was 0.16 ± 0.23 mm a year, the mean TZD was 0.52 ± 0.22 mm (range 0.05 to 1.24 mm), and the mean radius of TZS was 1.90 ± 0.12 mm (range 1.59 to 2.31 mm).

### Association between axial elongation and parameters

**Fig. 2:**
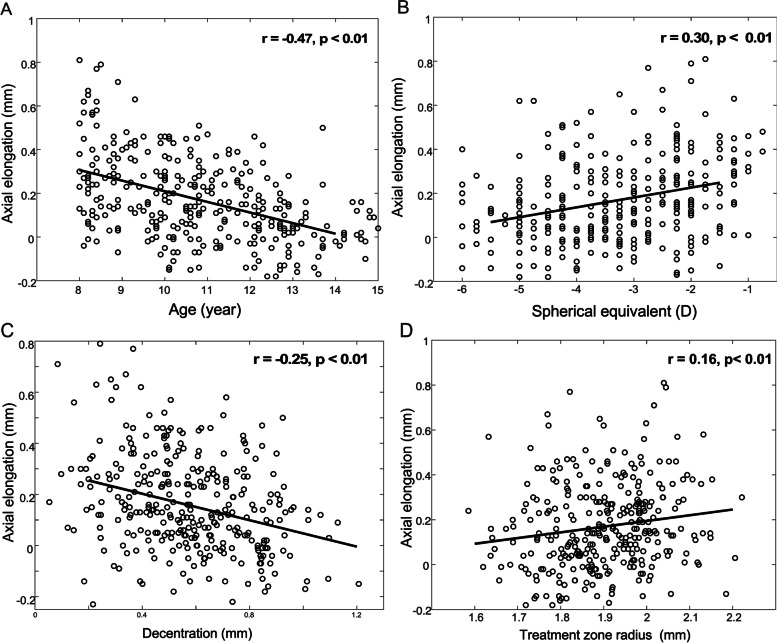
Axial elongation vs. baseline information (**A**) age and (**B**) spherical equivalent. Axial elongation vs. topography parameters (**C**) treatment zone decentration and (**D**) treatment zone size

The association between axial elongation and the parameters were first analyzed by univariate linear regression. Axial elongation was significantly associated with age and baseline SE (Fig. 2A, B). Axial elongation became slower in older children (*P* < 0.01) and children with greater baseline SE (*P* < 0.01). Axial elongation was significantly associated with TZS (Fig. 2D) and TZD (Fig. 2C). Axial elongation became slower in children with a smaller TZS (*P* < 0.01) or a larger TZD (*P* < 0.01). The corneal flat-K and steep-K were not associated with axial elongation (both *p* > 0.05).

### Classification based on decentration and treatment zone size

**Fig. 3:**
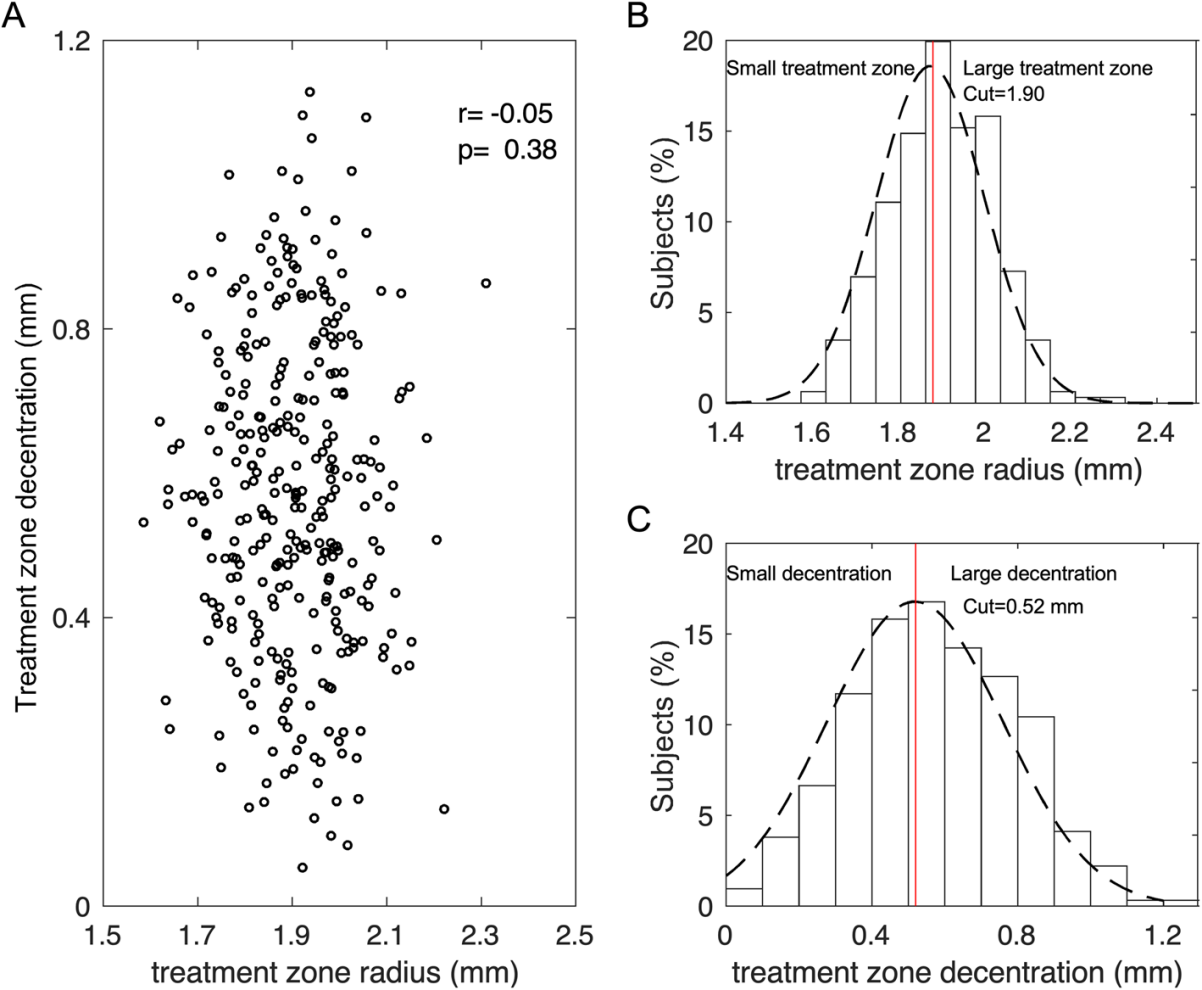
Treatment zone size (TZS) and treatment zone decentration (TZD). **A** correlation between TZS and TZD, **B** distribution of TZS, and **C** distribution of TZD

There was no significant correlation between TZD and TZS (*r* = − 0.05, *P* = 0.38, Fig. 3A). Overall, the TZS was normally distributed (grey dotted line in Fig. 3B). The children were divided into two groups at the mean of the normal distribution (red vertical line in Fig. 3B): small TZS (r ≤ 1.90 mm) and large TZS (r > 1.90 mm). After grouping, there was no significant difference in age and baseline SE between the small TZS group and large TZS group (Both *p* > 0.05). The TZD data also followed a normal distribution (Fig. 3C), and children were divided at the mean of the normal distribution into two groups: small TZD (≤ 0.52 mm) and large TZD (> 0.52 mm). There was no significant difference in age and baseline SE between small TZD and large TZD group (Both p > 0.05). All children were categorized using these four combinations of TZS and TZD.

### Association between axial elongation and baseline parameters both in TZD and TZS groups

**Fig. 4:**
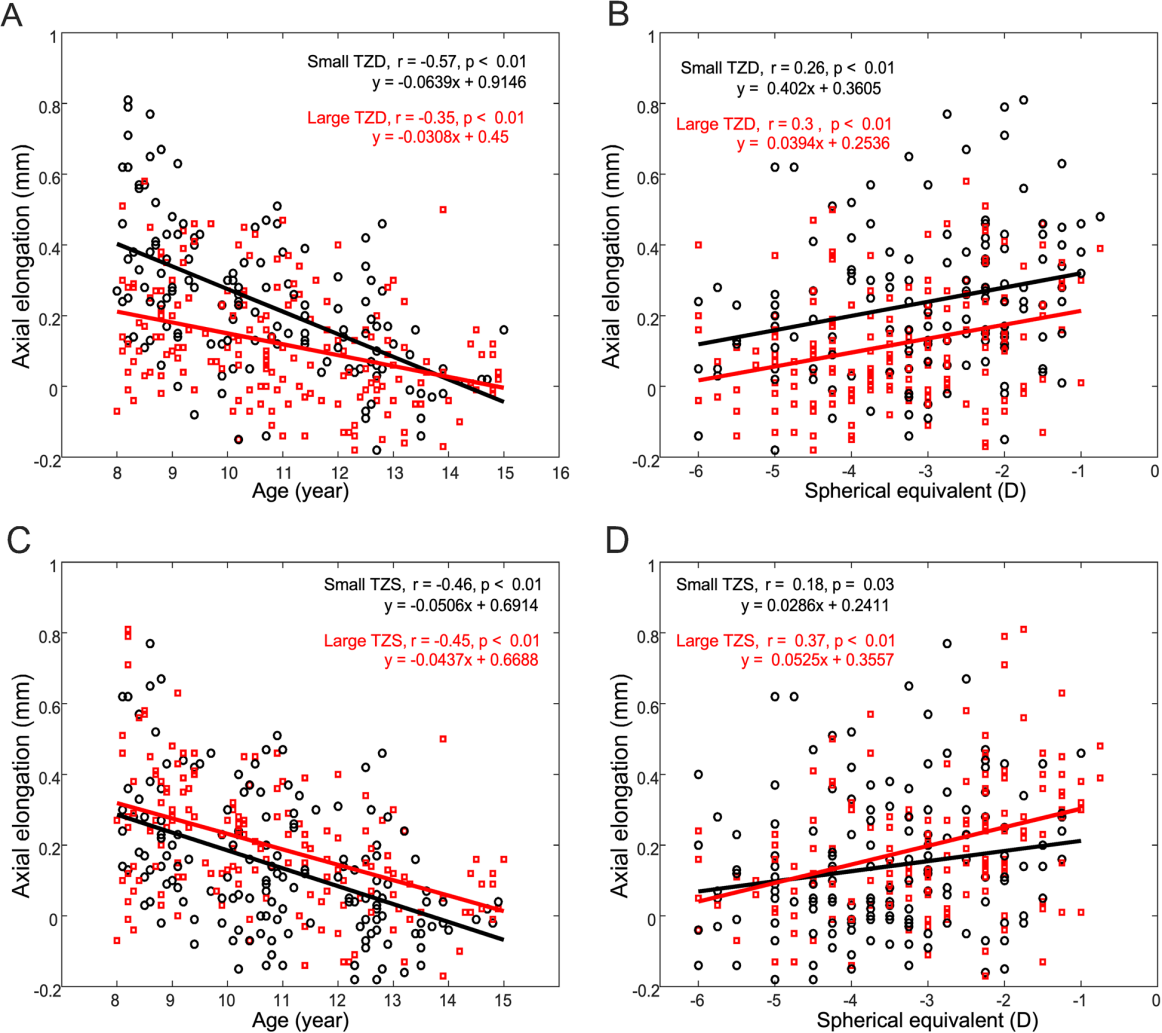
Axial elongation in function of baseline age and baseline SE of all children in both TZD and TZS groups. Small TZD vs. Large TZD (**A**) age and (**B**) spherical equivalent. Small TZS vs. Large TZS (C) age and (D) spherical equivalent

Figure 4 shows the association between Axial elongation and baseline age and baseline SE after subgroup both in TZD and TZS groups. A negative correlation is observed, demonstrating that older children show smaller axial elongation both in TZD and TZS groups (Fig. 4 A, C). A positive correlation showing that higher baseline SE display a smaller axial elongation both in TZD and TZS groups (Fig. 4B, D).

### Axial elongation versus treatment zone decentration and treatment zone size

**Fig. 5:**
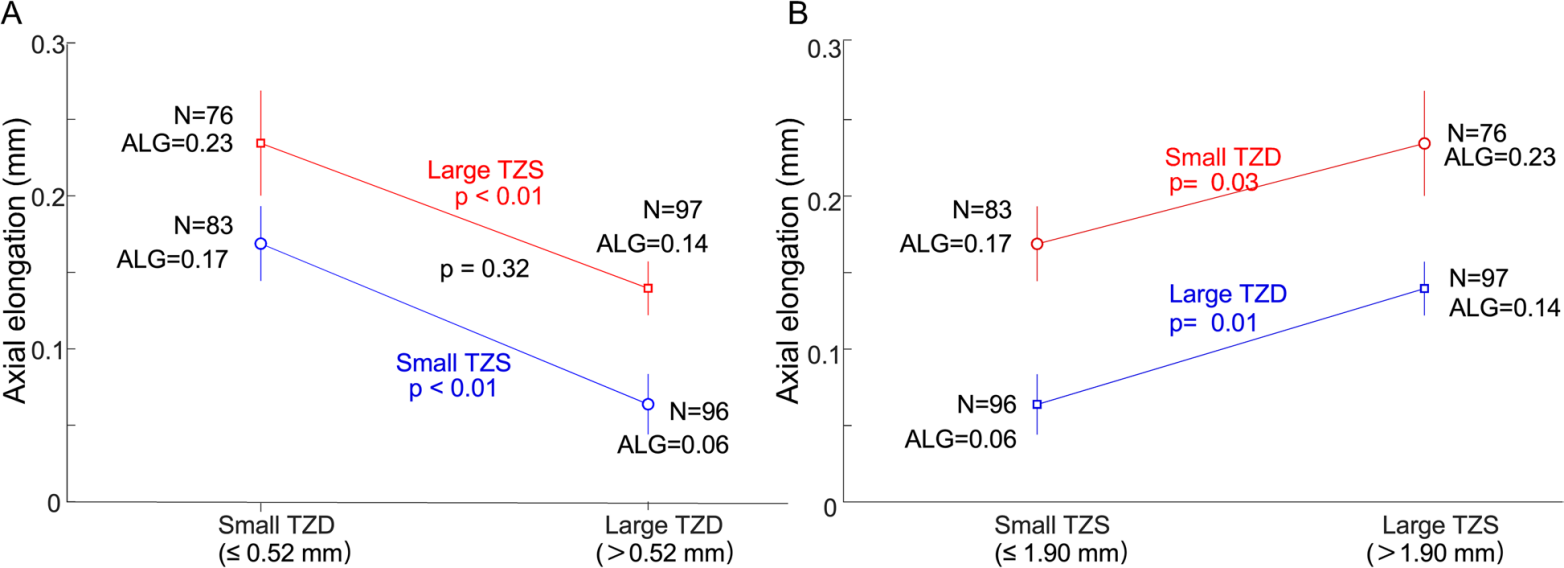
Axial elongation versus TZS and TZD. **A** Blue = Small TZS. Red = Large TZS. *P* values in red and blue represent comparisons across groups with different TZS. P values in black represent comparisons between small TZD with small TZS and large TZD with large TZS. **B** Blue = Large TZD. Red = Small TZD. P values in red and blue represent comparisons across groups with different TZD

In groups with either small TZS or large TZS, the axial elongation was significantly negatively associated with TZD (Fig. 5A, *P* < 0.01). In groups with either small TZD or large TZD, myopia control was improved with a decrease in TZS (Fig. 5B, *P* = 0.03, *P* = 0.01). However, there was no significant difference in axial elongation between children who had both small TZS and small TZD and those who had both large TZS and large TZD (Fig. 5A, *P* = 0.32).

To explore the association of axial elongation with the factors age, baseline SE, flat-K, steep-K, TZD, and TZS, a multiple regression analysis was applied in a stepwise manner. Multiple regression analysis revealed that axial elongation was significantly correlated with age, SE, and TZD (Table 1).

**Table 1:** Multivariable regression analysis showing the association between axial elongation and age, spherical equivalent, treatment zone decentration

Variables	Slope	***P*** value	95% CI
Age	−0.048	*P* < 0.01	− 0.060 to − 0.037
Spherical Equivalent	0.036	*P* < 0.01	0.018 to 0.054
Treatment Zone Decentration	−0.182	*P* < 0.01	− 0.283 to − 0.083
Overall model	R^2^ = 0.2607	*P* < 0.01	

## Discussion

In current study, children with smaller TZS and larger TZD benefited from a greater slowing of myopia progression with 12 months orthokeratology. Multiple regression showed that initial age, baseline SE and TZD were significantly associated with axial elongation.

### Initial age and baseline spherical equivalent

Many studies have examined factors that influence axial elongation during orthokeratology treatment, and initial age and baseline SE have been reported to be critical factors in affecting axial elongation. In our study, both initial age and baseline SE were significantly correlated with axial elongation in multiple linear regression (Table 1), with older children and greater baseline SE associated with smaller axial elongation. Other studies have reported differing associations [11]. Zhong et al. reported that initial age did not affect axial elongation on 32 children aged 9–14 years old in a 24-month follow-up study [34]. In contrast, Rubido et al. reported that initial age is significantly negatively correlated with axial elongation [35], and Wang et al. demonstrated that older initial age at the onset of orthokeratology lens wear was correlated with reduced axial elongation in myopic children [36]. The current study agreed with the results of the studies by Wang et al. and Rubido et al. and demonstrated that initial age significantly affected myopia control in orthokeratology treatment.

The association between axial elongation and baseline SE has also been debated [11]. In studies that reported a significant negative correlation between axial elongation and baseline SE, the subjects had a wider baseline SE range, typically between − 6.0 and − 1.0 D [36, 37]. In studies that reported a lack of association between baseline SE and axial elongation, the subject’s baseline SE was in a limited middle range, mostly between − 4.0 and − 1.0 D [8, 38]. In our current study, we found that older initial age and greater baseline SE were beneficial in slowing the progression of myopia in children receiving orthokeratology treatment for twelve months follow up.

### Treatment zone decentration

TZD is a common phenomenon in orthokeratology clinical practice and is difficult to avoid. Many factors may contribute to TZD, such as corneal asymmetry, lens fitting, lens diameter, corneal astigmatism [11, 39–43].Smaller lens diameter and greater corneal astigmatism are more likely to result in lens offset and TZD [24, 41, 42].Traditional orthokeratology guidelines encourage clinicians to pursue perfect centering with a bull’s-eye pattern during orthokeratology lens fitting. In traditional orthokeratology practice, there is no clear guideline on how much TZD should be allowed and how hard one should push for perfect centering.

In the current study, the mean TZD was 0.52 ± 0.22 mm (range 0.05–1.24 mm) which was in line with previous studies. Li et al. reported a mean TZD of 0.68 ± 0.35 mm (range 0.05–1.49 mm) from a study of 106 subjects [26], and Chen et al. reported a mean TZD of 0.72 ± 0.26 mm (range 0–1.34 mm) [41].Chen et al. reported a mean TZD of 0.64 ± 0.38 mm (range 0.13 to 1.78 mm) [24]. In the current study where 352 subjects were analyzed, we found that the TZD was significantly negatively correlated with axial elongation (Fig. 2C, *P* < 0.01), and our study, therefore, provides evidence to clarify the relationship between TZD and the slowing of myopia progression with orthokeratology treatment for twelve months follow-up. Children increased TZD was beneficial in controlling myopia, with relatively larger TZD associated with smaller axial elongation (Fig. 5A). Nevertheless, we state that the trends between TZD and axial elongation would need a longer period of study to confirm. We do not suggest deliberate decentration of the orthokeratology lens, as large TZD can cause visual discomfort, such as ghosting and visual fatigue [44, 45]. It is important to identify subjective sensations caused by TZD and then decide whether it is necessary to adjust the lens parameters.

### Treatment zone size combined with treatment zone decentration

TZS and TZD were two previously neglected factors for myopia control effectiveness, compared with the known factors such as initial age and SE at baseline. We found that in subjects with smaller TZS, larger TZD was associated with the smallest axial elongation (0.06 mm a year, Fig. 5A). In subjects with larger TZS, smaller TZD was associated with the largest axial elongation (0.23 mm a year, Fig. 5A). There was no axial elongation difference between children with both smaller TZS and smaller TZD and those with both larger TZS and larger TZD (*P* = 0.3212, Fig. 5A). When multiple linear regression was used to control for the contribution from initial age and baseline SE, only TZD was significantly associated with axial elongation. The reason for TZS being excluded by multiple regression may be that only one design of orthokeratology lens was used in the current study (Euclid, back optic zone diameter is 6.2 mm), which may result in a large range of TZS (radius 1.59 to 2.31 mm) with continuous boundary. Two different orthokeratology lens designs (different in TZ diameter and same total lens diameter) should be included in further research. Multiple linear regression is necessary to identify the factors that independently influence axial elongation.

### The potential mechanism

The mechanism of orthokeratology in control of myopia progression is still not clear. We hypothesized that orthokeratology induces myopia defocus in relative peripheral refractive error interfering the axial elongation pattern as “peripheral refraction theory”, which has been recognized by most researchers [46–48]. Cho et al. [49] hypothesized that the greater the corneal reshaping effect, the greater peripheral myopic defocus, the higher the regulation efficacy in retarding myopia progression. Yang et al. [50] suggested that areal summed corneal power shift (ASCPS) in a 4 mm area was a potential predictor of axial elongation in orthokeratology treatment. Wang et al. [22] agreed that a maximum value of post-treatment corneal relative power (PCRP) resulted in a higher probability of effective axial elongation control. Zhong measured the relative corneal refractive power shift (RCRPS) in the nasal, temporal, and inferior axes and found that the maximum changes were negatively correlated with 2-year axial elongation [34]. With a decentered treatment zone or a smaller treatment size, the reverse zone which has positive RCRPS moves closer to the pupil center. This could lead to a larger summed RCRPS within a 4 mm area, which agrees with the study by Yang et al. [50]Given the same pupil size, TZD, and a smaller TZS, the summed RCRPS within the pupillary zone would be much greater in corneal power profiles, which would agree with the larger pupil size often associated with smaller axial elongation [21].Jaume et al. found [29] that smaller back optic zone diameter reduce plus power ring diameter and showed better axial elongation than larger back optic zone diameter with orthokeratology. However, a recently study, Paul et al. [30] found that reducing treatment zone diameter did not alter relative peripheral refraction with orthokeratology in a week. We speculate that a smaller TZ and a higher decentered one will move the mid-peripheral ring inside the pupil if it is of the appropriate size, inducing higher optical changes that may be beneficial for myopia control in the children receiving orthokeratology treatment. However, in current retrospective study we did not measure the pupil size and RCRPS directly, we will incorporate these factors in the further prospective study. Another potential mechanism may be that corneal shape asymmetry is increased with orthokeratology TZD. Corneal shape asymmetry increases higher-order corneal aberrations [19–21, 51]. Hiraoka et al. found that increased corneal coma was significantly associated with decreased axial elongation in orthokeratology treatment [25].More recently, Jason et al. found that positive spherical aberration associated with axial elongation in orthokeratology [20]. Jason et al. suggested that the potential role of HOA, particularly spherical aberration may as the possibly mechanism of axial elongation with orthokeratology [19].

### Limitations of the current study

There are several limitations to the current study. First, we suggest that TZS and TZD benefit the retinal myopic defocus, but we did not directly measure peripheral retinal defocus in this study. Second, we speculate that the myopic control with TZD might shift more PCRP into the pupillary area as a potential mechanism for orthokeratology, but we did not measure the children’s pupil. Third, we speculated that higher-order corneal aberrations were associated with myopia control, but we did not directly measure higher-order aberrations. Fourth, the method to quantification of TZD relative to the corneal center rather than pupil center may have a slight impact on the results.

## Conclusion

Children with relatively smaller TZS and larger TZD after Orthokeratology experienced slower axial elongation by the end of 12 months. This effect might be mediated by the induction of a greater amount of relative myopic defocus on the peripheral retina. Further studies are needed to assess whether change on lens designs increase efficacy for slowing progression of myopia.

## Data Availability

The datasets used and/or analyzed during the current study available from the corresponding author on reasonable request.

## Abbreviations

TZS: the treatment zone size
TZD: Treatment zone decentration
SE: Spherical equivalent
ASCPS: Areal summed corneal power shift
PCRP: Post-treatment corneal relative power
RCRPS: Relative corneal refractive power shift
